# The Little Courtesies of Life

**Published:** 1880-10

**Authors:** 


### HALL'S JOURNAL OF HEALTH.

Our Legitimate Scope is almost boundless: for whatever begets pleasurable and harmless feelings, promotes Health; and whatever induces disagreeable sensations, engenders Disease.

WE AIM TO SHOW HOW DISEASE MAY BE AVOIDED, AND THAT IT IS BEST, WHEN SICKNESS COMES, TO TAKE NO MEDICINE WITHOUT CONSULTING A PHYSICIAN.

### THE LITTLE COURTESIES OF LIFE.

In walking through the streets of Paris, one scarcely fails to be struck with the life, light, and animation which prevails everywhere, and seems to pervade almost every body and every thing. The traveller from murky London or anxious New York, or stiff, calculating, skinny Boston, feels himself to be in a new atmosphere, and before he is aware he is hurried along with the living tide of the Boulevards or Champs Elysees, a polite and smiling gentleman,—his own countenance so brightened up with a cheery gladsomeness and sunshine, that he would not know his own phiz if suddenly confronted with a mirror. Everywhere there are birds, and songs, and flowers, and smiles; at every turn there is such a seeming unaffected courtesy and polite deference that the most common person can scarce avoid coming to the conclusion that he is somebody, and he retires to his hotel with a lighter and more satisfied heart than he has had for many a long day, and places his head upon his pillow, well pleased with all the world. The Editor's reminiscences of beautiful Paris, in the palmy days of Louis Napoleon are all of flowers and sunshine. If sauntering in Union Park he took a seat on some vacant bench, the very next comer moved on the last two inches of the utmost extremity, in three cases out of four giving a view of his back; in sixteen seconds more he would be making numberless gyrations with his cane or boot toe on the gravel walk; if the bench happened to be on the flagging he would fix his eye on some spot and spit at it by the quarter; no cheerful flitting ever coming across that sad reflecting face even for the briefest moment, as if there were not a thought or a sympathy for any human being. Why not give time to gold and time to gladness too, and let each have its season: be serious if you please in Wall-street or behind the counter, but in the car or omnibus, or park or square, or church or promenade, let an inner joyousness light up the countenance, and let the smile of recognition of your brother man wake up new life whenever the eye falls upon that brother's countenance; it will seldom fail to light up a kindred gladness there, self-perpetuating all along glorious old Broadway, from Union square to the Battery; all of us would live the longer for it, and what is more, live the happier. I move that no vinegar cruet be allowed in Broadway until moon down. What right has any man to come up to me, without cause or provocation, when I am gladsomely strolling down town, with little Nell and Molly, each holding on to a forefinger, to turn my face into a tamarind? They will see it in a moment, and their little hearts will beat less joyously, until we get to the next candy shop These are little things it is true, but the mass of human enjoyment or sorrow is made up of these self-same little things. A writer well says:—

"The little things of life have far more effect upon character, reputation, friendship, and fortune than the heartless and superficial are apt to imagine. They are few indeed, however rough by nature, who are not touched and softened by kindness and courtesy. A civil word, a friendly remark, a generous compliment, an affable bow of recognition—all have an influence—while surliness, incivility, harshness and ill-temper naturally enough produce an effect exactly to the reverse. The American people, as a whole, are perhaps not remarkable for courtesy. They are so actively engaged in the bustle of life, in onward movements of commerce and trade, that they have little leisure to cultivate and practice those polished refinements, which are the results of education, of travel, and of enlarged intercourse with society. Nevertheless, we are not a discourteous people, and in the great cities the proprieties of manner, and the civilities of form are attended to with a commendable degree of exactness.

